# Enhancing Cellular Internalization of Single-Chain Polymer Nanoparticles via Polyplex Formation

**DOI:** 10.1021/acs.biomac.2c00858

**Published:** 2022-11-16

**Authors:** Naomi M. Hamelmann, Sjoerd Uijttewaal, Sry D. Hujaya, Jos M. J. Paulusse

**Affiliations:** Department of Molecules and Materials, MESA+ Institute for Nanotechnology and TechMed Institute for Health and Biomedical Technologies, Faculty of Science and Technology, University of Twente, P.O. Box 217, 7500 AE Enschede, The Netherlands

## Abstract

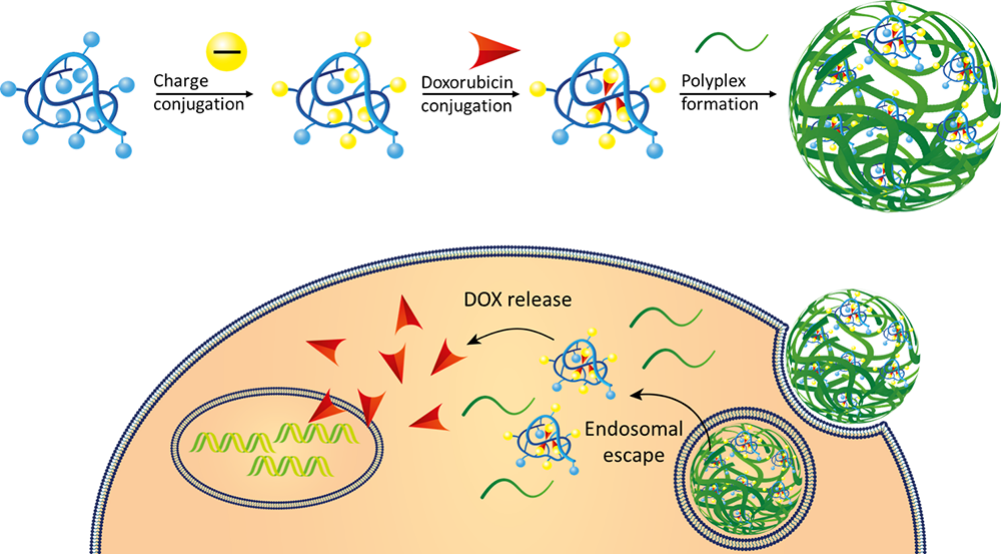

Intracellular delivery of nanoparticles is crucial in nanomedicine to reach optimal delivery of therapeutics and imaging agents. Single-chain polymer nanoparticles (SCNPs) are an interesting class of nanoparticles due to their unique site range of 5–20 nm. The intracellular delivery of SCNPs can be enhanced by using delivery agents. Here, a positive polymer is used to form polyplexes with SCNPs, similar to the strategy of protein and gene delivery. The size and surface charge of the polyplexes were evaluated. The cellular uptake showed rapid uptake of SCNPs via polyplex formation, and the cytosolic delivery of the SCNPs was presented by confocal microscopy. The ability of SCNPs to act as nanocarriers was further explored by conjugation of doxorubicin.

## Introduction

Nanoparticles (NPs) are highly modular materials that can be engineered to deliver therapeutics or imaging agents to specific locations in the body. This strategy is utilized to increase the efficacy of therapeutics and diminish side effects. Polymeric NPs provide ample opportunities in the field of controlled drug delivery, such as control over size,^1^ composition,^2^ and surface functionality,^3^ allowing encapsulation and conjugation of therapeutics.^4−7^ The size of NPs has been shown to strongly influence their biodistribution behavior.^8^ Particles smaller than 10 nm are prone to rapid clearance by the kidneys, whereas NPs above 200 nm in size tend to accumulate in the liver and spleen.^9^ NPs with sizes in the range of 10 to 200 nm have shown great variation in biodistribution behavior; for example, inorganic NPs of 10–15 nm in diameter have been shown to reach the brain more readily than larger-sized NPs.^10,11^ Also, deeper penetration has been shown for small NPs in poorly permeable hypovascular tumors.^7,12^ Further research on size-dependent cellular uptake indicates a general increase in cellular uptake for smaller-sized NPs.^13^ While various preparation strategies are well known for NPs above 50 nm, smaller NP systems such as dendrimers have more intricate synthetic routes.^14,15^ A different but appealing strategy to develop small NPs is intramolecular crosslinking of individual polymer chains, forming single-chain polymer nanoparticles (SCNPs).^16^ This class of NPs is characterized by monodisperse size distributions and the ability for easy scale up of their production.^16−19^ The SCNPs have unique sizes in the range of 5 to 20 nm, and their characteristics are highly dependable on the precursor polymer. Highly controlled functionalization of SCNPs can be achieved either before or after crosslinking of the polymer chains, and Palmans and co-workers have used pentafluorophenol (PFP) acrylate-based polymers to conjugate functional side chains onto the polymers, after which they are crosslinked into SCNPs.^20^ We previously reported the preparation of PFP-SCNPs from PFP-functional polymers that are intramolecularly crosslinked via a thiol-Michael addition and subsequently conjugated with various amines.^21^^,^^22^ Drug encapsulation of therapeutics including rifampicin,^5^ cisplatin,^23^ and vitamin B9^24^ has been demonstrated in SCNPs, leading the research of SCNPs toward the application of controlled drug delivery.

There are several barriers for NPs to overcome before reaching their specific target, and one of these is the cell membrane.^25,26^ The cellular uptake and the intracellular location of NPs are keys to the development of efficient nanocarriers. Although several studies have shown the uptake of SCNPs in cells, in most cases, the SCNPs remain predominantly trapped in endosomal structures.^5,27^ Research into the functionalization of SCNPs to direct them toward cytosolic delivery has been carried out through the addition of positive charges on the SCNP surface^28^ as well as through the addition of a dendritic transporter.^29^ However, for SCNPs without protonatable amines, cytosolic delivery remains challenging.^27^ For these SCNPs, cytosolic delivery has only been shown using physical strategies such as electroporation. Liu et al. incubated polyacrylamide-based SCNPs functionalized with oligo (ethylene oxide-*co*-propylene oxide), benzene-1,3,5-tricarboxamide, and catalytically active sites with HeLa cells and utilized electroporation for cytosolic delivery.^27^

Cationic polymers have commonly been used to form polyplexes with DNA^30−32^ and proteins^33−35^ to achieve cytosolic delivery. Poly(amido amine)s have been widely used in gene delivery.^36,37^ These peptidomimetic polymers contain amines, which can be protonated intracellularly through the proton sponge effect and present the ease of integrating disulfide moieties for biodegradability.^30,37,38^ pCBA-ABOL, a copolymer containing *N*,*N*-bis(acryloyl) cystamine and 4-amino-1-butanol, is degradable in the presence of glutathione and has shown promising gene transfection results.^30^

Here, we explore the use of a cationic, reducible polymer-based vector to traffic anionically charged SCNPs to the cytosol. In previous research, pCBA-ABOL has shown low toxicity and high transfection efficiencies,^30,39^ which make this polymer an interesting delivery tool for SCNPs. The polyplex formation of anionically charged SCNPs and pCBA-ABOL at various weight ratios is investigated by DLS and zeta potential measurements. The biocompatibility of the polyplexes is evaluated in Hela cells. Cellular uptake as well as intracellular location of fluorescently labeled pCBA-ABOL and SCNPs is investigated by confocal microscopy and flow cytometry. Doxorubicin (DOX) is conjugated onto anionic SCNPs, and cellular uptake via polyplex formation is explored in HeLa cells to evaluate the potential for intracellular drug delivery.

## Materials and Methods

### Materials

DMSO (anhydrous, 99.9%), poly(ethylene glycol) (PEGDA, *M*_n_ 258 g/mol), hydrazine monohydrate (98%), dimethylaminoetyl acrylate (DMAEA, 98%), tris(2-carboxyethyl)phosphine hydrochloride (TCEP, ≥98%), succinic anhydride (99,9%, Fluka), pyridine (>99%), *N*-methylisatoic anhydride (MIA, 90%), doxorubicin hydrochloride (>98%), *N*-(3-dimethylaminopropyl)-*N*′-ethylcarbodiimide hydrochloride, *N*-hydroxysuccinimide (98%), Dulbecco modified Eagle’s medium (DMEM), fetal bovine serum (FBS), penicillin–streptomycin (containing 10.000 units penicillin, 10 mg streptomycin mL^–1^), trypsin–EDTA solution (sterile filtered, BioReagent), phosphate buffered saline (PBS, pH 7.4), and resazurin sodium salt (BioReagent) were purchased from Sigma-Aldrich. Propidium iodide (PI), LysoTracker Red DND-99, 5-(4,6-dichlorotriazinyl) aminofluorescein (DTAF), and ActinRed 555 ReadyProbes Reagent (Rhodamine phalloidin) were purchased from ThermoFischer Scientific. All chemicals were used without purification except if stated otherwise. SnakeSkin dialysis tubing (10 K MWKO) was from ThermoFisher Scientific, and PD-10-desalting columns were purchased from GE Healthcare. pCBA-ABOL was synthesized following our earlier literature procedure.^30^ Dynamic light scattering (DLS) and zeta potential measurements were performed on a Malvern Instrument Zetasizer ZS in 10 mM NaCl solution.

### Glycerol SCNP Synthesis

Glycerol SCNPs were formed as previously reported.^5^ In brief, copolymer p(XMA-SMA) (XMA:SMA 1:9, 500 mg) was dissolved in 10 mL of DMSO and the thiol moieties on the xanthate groups (0.35 mmol eq. thiol monomer) were deprotected by the addition of hydrazine (34.4 μL, 0.7 mmol, 2 eq.). The deprotected copolymer was filtered and then added dropwise to a dilute solution of carbonate–bicarbonate (CB) buffer containing poly(ethylene glycol (PEGDA, 258 g/mol, 86.8 μL, 0.35 mmol, 1 eq.) and TCEP (18.5 mg, 0.06 mmol, 0.2 eq.) to induce the intramolecular crosslinking via thiol-Michael addition. *N*,*N*-Dimethylaminoethyl acrylate (DMAEA, 1.9 mL, 12.4 mmol) was added to end-cap the remaining thiols. The resulting particles were purified by dialysis and isolated by lyophilization (∼300 mg).

### Succinic Anhydride Conjugation

Glycerol SCNPs were functionalized as previously described.^40^ In brief, glycerol SCNPs (40 mg, 0.2 mmol in glycerol units), succinic anhydride (20.8 mg, 0.2 mmol, 1 eq.), and pyridine (16.8 μL, 0.2 mmol, 1 eq.) were dissolved in 7 mL of DMSO and stirred at room temperature overnight. The particles were purified by dialysis and obtained by lyophilization (∼20 mg).

### DTAF Labeling of Anionic SCNPs

SCNPs (20 mg, 0.1 mmol in glycerol units) were dissolved in 5 mL of CB buffer, and 0.9 mg of DTAF (0.002 mmol, 0.02 eq.) was added. The solution was stirred overnight. The fluorescently labeled particles were purified using a PD-10 column. The particles were obtained by lyophilization (∼10 mg).

### MANT Labeling of pCBA-ABOL

The pCBA-ABOL polymer was prepared as previously described^41,42^ and labeled by dissolving 100 mg (0.27 mol per alcohol moieties) in 10 mL of DMSO and adding 48.7 mg of MIA (0.27 mol, 1 eq.) to the solution. The reaction was filtered and dialyzed after 1 h of stirring. The labeled pCBA-ABOL was obtained after lyophilization (∼70 mg).

### Doxorubicin Conjugation onto Anionic SCNPs

Anionic SCNPs (DTAF) (anionic SCNP-hP1(31) DTAF, 20 mg) were dissolved in 2.6 mL of PBS (pH 7.4) at 7.5 mg/mL, and EDC (8.9 mg, 0.5 eq.) and NHS (5.6 mg, 0.5 eq.) were added to the solution, which was stirred for 30 min. Then, 108 μL of DOX (5 mg/mL, 0.01 eq.) was added to the solution and stirring was continued for 3 h, protected from light. The SCNPs were purified using a PD-10 column and lyophilized, yielding 17.2 mg of DOX-SCNPs. Absorbance measurements by UV–Vis of free Dox in PBS were utilized to calculate a calibration curve and to compare to DOX-SCNPs in PBS to determine the final drug loading of the particles.

### DOX Release from DOX-SCNPs

DOX-SCNPs were incubated in cell lysate, and samples were taken after 2 and 24 h. The samples were centrifuged at 12000 rpm for 20 min, and the UV–Vis of the supernatant was measured.

### Cell Viability

Hela cells were cultured in 96-well plates by adding 100 μL of DMEM medium containing 7.5 × 10^3^ cells per well and incubated overnight at 37 °C in a humidified 5% CO_2_-containing atmosphere. Polyplexes were prepared using stock solutions of 1 mg/mL SCNPs and 6 mg/mL pCBA-ABOL, the SCNPs were diluted to 10 μg/mL (final concentration) in Milli Q, and pCBA-ABOL was added to 6.25, 12.5, 25, 50, 75, and 100 charge weight ratios to a volume of 100 μL, and after 10 min incubation at room temperature, 200 μL of medium was added. As an example for polyplex solution of 6.25 ratio, 3 μL of SCNP stock solution was diluted in 95.6 μL of Milli Q and 1.35 μL of pCBA-ABOL stock solution was added, and after 10 min, 200 μL of medium was added. The polyplex solution was added to the Hela cells, and after aspiration of the medium, by adding 100 μL per well, each sample was measured in triplicate. The reference cells were incubated with the medium, and a control of SCNPs 10 μg/mL was used. After 4, 6, or 8 h, the polyplex medium was replaced by a fresh medium. The cell viability was analyzed after 24 h by adding resazurin solution to each well (440 μM) and was incubated for 4 h. The fluorescence signal was measured by an Enspire plate reader with excitation and emission wavelengths of 560/590. Statistical analysis (*n* = 9) was performed utilizing one-way analysis of variance (ANOVA) with Tukey post hock analysis. The classifications of the differences were reported as follows: significant (*p* < 0.05), very significant (*p* < 0.01), and extremely significant (*p* < 0.001).

### CLSM of Polyplex Uptake

On a 96-well plate, Hela cells were seeded at 7.5 × 10^3^ cells per well in 100 μL medium and incubated overnight. Polyplexes were prepared as earlier described, using SCNPs labeled with DTAF and pCBA-ABOL with or without the MANT label, and added to the cells at 10 μg/mL SCNP concentration in 100 μL. The polyplexes were incubated for 3 h. Afterward, the cells were fixated with 4% PFA solution and permeabilized with 0.1% Triton-X. Subsequently, the cells were stained with Actin staining for 30 min and washed and stored in PBS. For the samples stained with lysosome staining, the Hela cells were subsequently subjected to a 3 h particle incubation, which were also incubated with lysosome staining, before the cells were fixated and stored in PBS. The internalization of polyplexes was examined using a Nikon confocal microscope A1, equipped with the following laser wavelengths: 375, 488, and 561 nm.

### FACS of Polyplex Uptake

Hela cells were seeded in a 48-well plate at 30 × 10^3^ cells per well in 300 μL medium and incubated overnight. Sample solutions of polyplexes were prepared as earlier reported, and 300 μL of each sample was added per well, and as a control, 10 μg/mL SCNPs were used. The polyplexes were incubated for 1, 3, and 6 h, and then, the cells were washed with PBS and harvested with trypsin. The resulting cell pellet was resuspended in 300 μL of PBS. For the flow cytometry measurements, samples were additionally incubated with PI stain to analyze the cell viability. The FACS measurement was performed with a BD Bioscience FACS Aria II using excitation and emission filters of 375–450/30, 488–530/30, and 630/30 nm.

## Results

Glycerol SCNPs were prepared as earlier reported^5^ by slow addition of the copolymer to a crosslinker solution (see Figure S1). The intramolecular chain collapse was analyzed by size exclusion chromatography, presenting a smaller hydrodynamic radius for the SCNP compared to the precursor polymer. A particle size of 8.4 nm ± 1.3 nm was determined by DLS (Figure S2). To induce negative surface charges on the SCNPs, succinic anhydride was conjugated onto the alcohol moieties of the glycerol units shown by ^1^H NMR in Figure S3.^40^ The surface charge of the resulting negative SCNP (SCNP^–^) decreased to −43.3 mV compared to the −20.5 mV of the glycerol SCNP (see Figure S4). An important aspect of this post-formation functionalization strategy is that the surface functionalization does not significantly change the SCNP size (see Figure S5).

**Figure 1:**
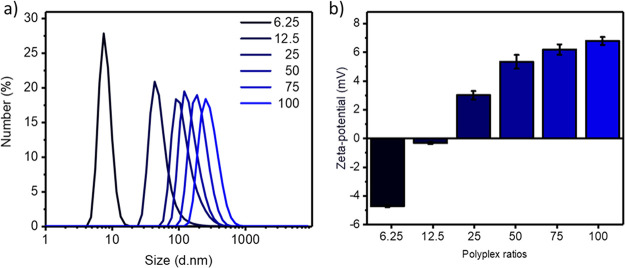
(a) Size of polyplexes with increasing w/w ratios measured with DLS by number and (b) surface charge of polyplexes with increasing w/w ratios.

**Figure 2:**
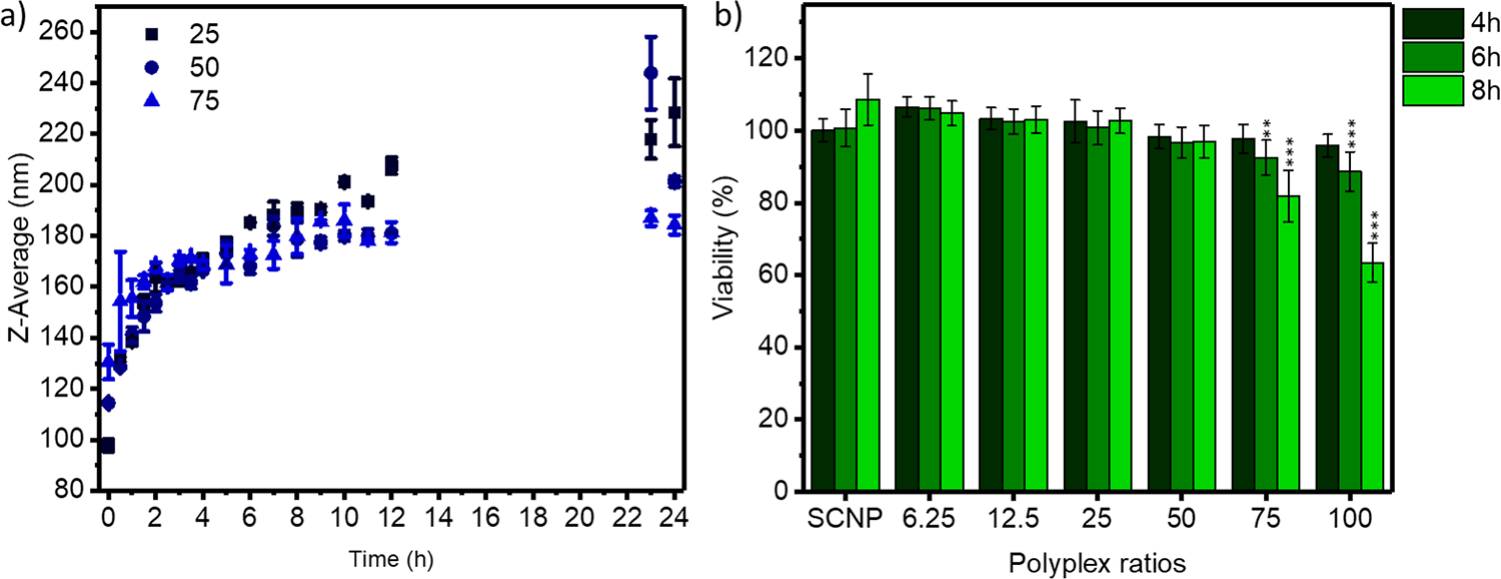
(a) Stability of polyplexes’ ratios 25, 50, and 75 measured by DLS. (b) Viability of Hela cells after incubation with SCNPs and polyplexes at various w/w ratios, with no significant (n.s.) decrease in viability compared to the reference.

pCBA-ABOL, containing protonable tertiary amines in the backbone, was mixed with SCNP^–^ to obtain polyplexes with increasing weight ratios ranging from 6 to 100 w/w pCBA-ABOL/SCNP^–^. The size of the polyplexes at different weight ratios was analyzed by DLS. Diameters of the formed polyplexes are presented in Figure 1a. At the 6 w/w polymer/SCNP ratio, the measured size is comparable to the size of bare SCNP^–^, while at the 12 w/w polymer/SCNP ratio, small-sized polyplexes (54 ± 11 nm) with a wide particle size distribution formed, as shown in the intensity plot in Figure S6. The sizes of the polyplexes increase continuously from 25 to 100 w/w polymer/SCNP ratios, which relates to the addition of higher amounts of pCBA-ABOL. Zeta potentials of the polyplexes are depicted in Figure 1b. The negative surface charge of the polyplex with the 6 w/w polymer/SCNP ratio confirms the DLS results, indicating that no stable polyplexes are formed. At the 12 w/w polymer/SCNP ratio, the zeta potential is around zero, while the higher ratios display positive zeta potentials. Correlating with the increase in size, the surface charges of 25 to 100 w/w polymer/SCNP ratios increased continuously as well. The increase in zeta potential flattens as higher ratios are reached, indicating a saturation of the charges at the surface of the polyplexes. The stability of polyplexes in aqueous solution was evaluated over time, revealing swelling of the polyplexes, as shown in Figure 2a. During the first 4 h, swelling is rapid and slows down afterward, and for the polyplex with the 25 w/w polymer/SCNP ratio, the particle size doubled within 10 h from 100 to 200 nm.

PCBA-ABOL has shown potential in the intracellular delivery of genetic cargoes as well as proteins.^30,43^ To adapt this strategy for the delivery of SCNPs, the cytotoxicity of polyplexes containing SCNPs was first evaluated in Hela cells. In earlier literature reports, the toxicity of polyplexes based on pCBA-ABOL is generally evaluated by short incubation of cells with the polyplexes (e.g., 1 h), after which the medium is changed with fresh medium and cytotoxicity is evaluated after a total of 24 h, resulting in cell viabilities of >80% for all test ratios with pCBA-ABOL.^30^ Here, polyplexes were incubated for 4 h and subsequently incubated with fresh medium for a total of 24 h incubation (see Figure 2b). No significant decrease in cell viability compared to the reference was observed for any polyplex ratio. Longer incubation times were also tested for the polyplexes by incubating the polyplexes for up to 6 and 8 h before the medium was changed. Cell viability only decreases to below 80% for the 100 w/w polymer/SCNP ratio at 8 h incubation, and the toxicity is induced by pCBA-ABOL. In the literature, higher toxicities at shorter incubation times have been reported,^30^ indicating that the polyplexes formed with the SCNPs are favorable in terms of biocompatibility. Sprouse and Reineke investigated the toxicity and transfection efficiency of glycopolymers containing primary and tertiary amines and showed an increase in toxicity with higher tertiary amine contents.^44^ These results indicate that the toxicity induced by the polyplexes at a high pCBA-ABOL content is induced by the tertiary amines.

**Figure 3:**
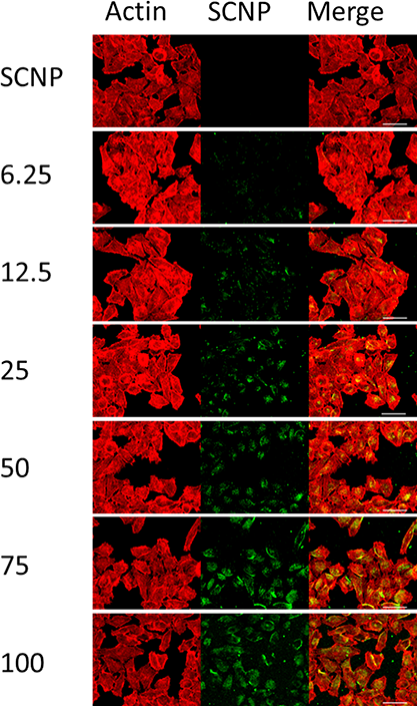
Confocal laser scanning microscopy images of Hela cells incubated with SCNPs and polyplexes with increasing w/w ratios for 3 h. Actin is stained in red, and SCNPs are labeled with a green fluorescent label (scale bar: 50 μm).

The intracellular delivery of SCNP^–^ via polyplexes was evaluated next by confocal laser scanning microscopy (CLSM). DTAF-labeled SCNPs were utilized in polyplex formation to be able to follow the SCNPs intracellularly. Polyplexes at various ratios were incubated with Hela cells for 3 h at an SCNP^–^ concentration of 10 μg/mL, and subsequently, Actin staining was utilized to visualize the Hela cells. However, CLSM did not reveal any signal of SCNP^–^ in the Hela cells after 3 h incubation (see Figure 3). Generally, SCNPs are incubated for prolonged times and higher concentrations are required for confocal images; Kröger et al. used glycerol SCNPs at a concentration of 500 μg/mL for 20 h.^5^ At the 6 w/w polymer/SCNP ratio, barely any SCNP^–^ signal is observed, which is in line with the earlier reported DLS and zeta potential results as this ratio has an overall negative surface charge. HeLa cells incubated with polyplexes with a 12 w/w polymer/SCNP ratio and higher ratios express stronger signals for SCNPs. Interestingly, polyplexes with a 25 w/w polymer/SCNP ratio and higher ratios display a signal for SCNP^–^ throughout the cells. The increase in uptake is likely due to the increased positive charge and tertiary amines introduced by the amount of pCBA-ABOL in the polyplexes. The proton sponge effect provided by the tertiary amines becomes strong enough at a 25 w/w polymer/SCNP ratio to achieve the endosomal release of SCNPs into the cytosol.

To quantify the cellular uptake of SCNPs, further flow cytometry (FACS) measurements were conducted. The uptake of SCNPs was analyzed after 1, 3, and 6 h incubation, and signals from both the fluorescently labeled SCNP^–^ and pCBA-ABOL were evaluated (see Figure S7). SCNP^–^ without pCBA-ABOL displays barely any signal after 1 h incubation and does not significantly increase with longer incubation times. After 1 h incubation, the polyplexes show an increase in SCNP^–^ signal in the Hela cells with increasing polyplex ratios. This trend is observed at each time point and is in line with the confocal microscopy results. The signal of pCBA-ABOL in HeLa cells increases with higher polymer/SCNP ratios at 1 h as well. After 3 h incubation, the SCNP signal do not increase compared to that after 6 h, while the signal of pCBA-ABOL increases. The significant swelling of the polyplexes within 4 h in aqueous solutions might result in less SCNP uptake over prolonged incubation times, while pCBA-ABOL, which is no longer complexated, is able to enter the cells more freely.

**Figure 4:**
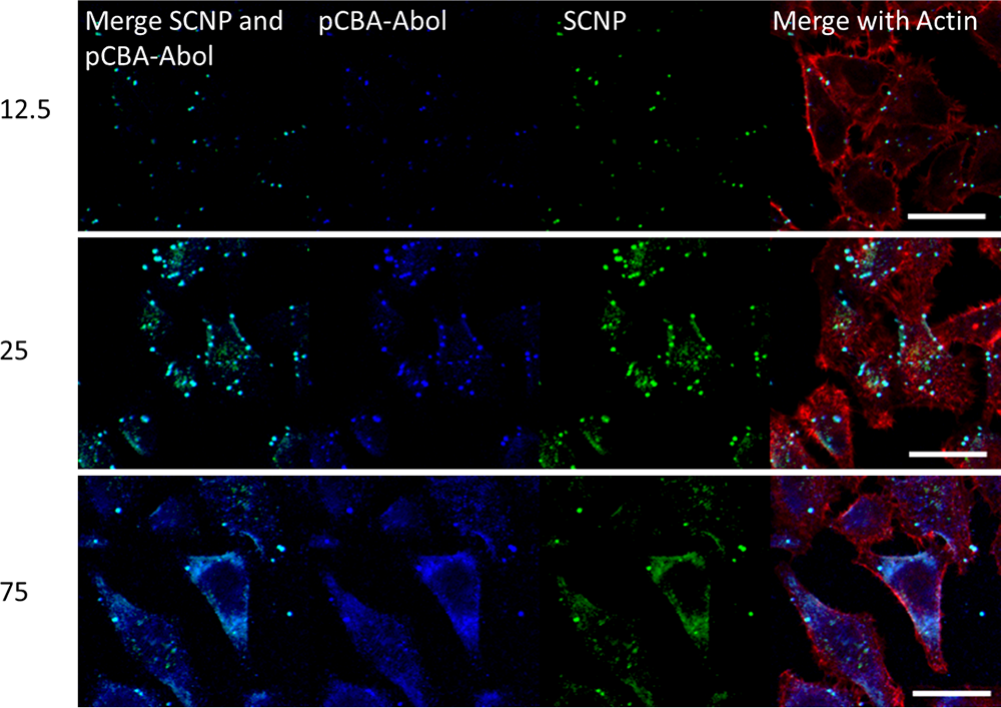
CLSM images of Hela cells incubated with polyplexes with selected w/w ratios to highlight the intracellular location of SCNPs and pCBA-ABOL. Actin is stained in red, and SCNPs are labeled with a green fluorescent dye and pCBA-ABOL with a blue fluorescent dye (scale bar: 25 μm).

The intracellular location of the SCNP^–^ with 12, 25, and 75 w/w polymer/SCNPs are depicted in Figure 4. Meanwhile, for the low polymer/SCNP ratio of 12 w/w, SCNP^–^ remains inside the vesicular structures; at a polymer/SCNP ratio of 25, a diffuse signal from the cytosol is observed, in addition to the vesicular signal. The endosomal escape is attained by the proton sponge effect, and these results indicate that there is a critical amount of pCBA-ABOL containing tertiary amines needed in the polyplexes to reach the cytosol. At the higher ratio of 75 w/w polymer/SCNP, the SCNP^–^ signal is shown throughout the cells, although not penetrating the cell nuclei, pointing to successful cytosolic delivery. Interestingly, the blue MANT signal from pCBA-ABOL colocalizes strongly with the SCNP^–^ signal in all three cases and is also not observed in the nuclei. The toxicity of polyplexes commonly increases with higher N/P ratios.^45^ Lower ratios are therefore favorable for improved biocompatibility. To confirm the intracellular location of SCNP^–^, lysosome staining was used for the HeLa cells (see Figure S8). This shows that the colocalization of SCNPs with lysosome staining decreases upon increasing the polymer/SCNP ratio, while the cytosolic delivery of SCNPs is enhanced.

**Figure 5:**
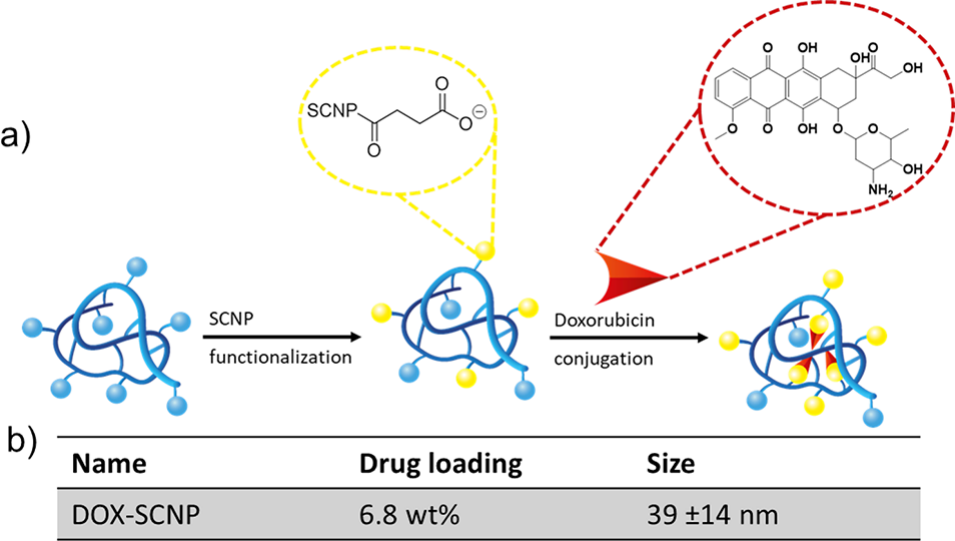
(a) Reaction scheme of DOX conjugation onto carboxylic acid groups of SCNPs. (b) Table presenting the drug loading and size of DOX-SCNPs.

The ability of SCNPs to act as nanocarriers in controlled drug delivery was assessed by conjugating doxorubicin (DOX), a powerful anticancer drug, onto the anionic SCNPs (see Figure 5).^46^ Successful conjugation of DOX onto the carboxylic acid moieties of the SCNPs was shown by the increase in surface charge upon DOX conjugation (see Figure S9). A drug loading of 6.8 wt % was measured by UV–Vis, and a particle diameter of 39 ± 14 nm was shown by DLS (shown in Figures S10 and 11).

**Figure 6:**
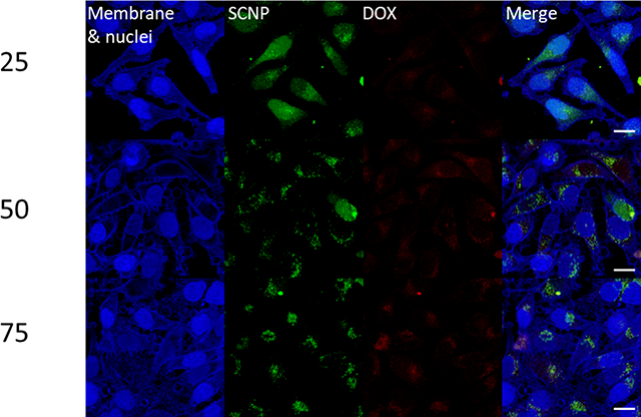
CLSM images of Hela cells incubated with polyplexes with selected w/w ratios and DOX-SCNPs. Cell nuclei and membranes are stained in blue, SCNPs have a green fluorescent label, and DOX signal is in red. The scale bar is 20 μm.

The release of DOX was studied by UV–Vis after incubation in cell lysate, and after 2 h of incubation, no signal was visible in the supernatant. However, after 20 h, the UV–Vis spectra show a signal at 487 nm corresponding to free DOX (see Figure S12). Intracellular drug delivery was evaluated by incubating HeLa cells with fluorescently labeled DOX-SCNPs in polyplexes with weight ratios of 25, 50, and 75. The cells were subsequently stained using membrane and nuclei staining in blue, as shown in the control images in Figure S13. The CLSM images show the polyplex-mediated cellular uptake of SCNPs and DOX after 4 h incubation, with colocalized signals from DOX and SCNPs (see Figure 6). Further evaluation revealed that higher SCNP concentrations were required for DOX to lower the cell viability. However, pCBA-ABOL also displays cytotoxicity at high concentrations and prolonged incubation times. Therefore, DOX-SCNP was also evaluated as nanocarriers, showing a significant decrease in viability after 72 h incubation at 50 and 100 μg/mL (see Figure S14).

## Conclusions

We present here a strategy to strongly enhance the internalization of negatively charged SCNPs within short incubation times in Hela cells via the formation of polymer/nanoparticle polyplexes. Importantly, this strategy highlights the ability of directing the location of SCNPs in cells based on the employed polymer/SCNP ratios. Polymer/SCNP ratios of 25 and 50 w/w display increased internalization of SCNPs over the course of 3 h, with the cytosolic delivery of SCNPs, while still remaining non-toxic, boding well for their use in biomedical applications. This approach allows for the introduction of SCNPs into the cell cytosol without the need for specific transporter molecules on the particle surface. Successful conjugation of DOX onto the SCNPs and concomitant drug release were demonstrated. In the future, research into utilizing positively charged polymers with a lower toxicity such as shown with polymers containing guanidine moieties will improve the biocompatibility of the polyplexes at high w/w polymer/SCNP ratios.

## Supporting Information Available

Experimental details, DLS data, ^1^H NMR spectra, zeta potential data, flow cytometry data and confocal microscopy images, and UV–Vis data regarding drug conjugation and release (PDF)
