# Supplementary material for: Enhancing Cellular Internalization of Single-Chain Polymer Nanoparticles via Polyplex Formation
Source: Biomacromolecules. 2022 Nov 16;23(12):5036–42. doi: 10.1021/acs.biomac.2c00858 (PMC9748935; doi:10.1021/acs.biomac.2c00858)
Supplement: Supplementary file 1 — bm2c00858_si_001.pdf [file bm2c00858_si_001.pdf]

Supporting Information

Enhancing Cellular Internalization of Single-Chain Polymer Nanoparticles via Polyplex Formation

Naomi M. Hamelmann,[a] Sjoerd Uijttewaal,[a] Sry D. Hujaya[a] and Jos M. J. Paulusse*[a]

[a] Department of Molecules and Materials, MESA+ Institute for Nanotechnology and TechMed Institute for Health and Biomedical Technologies, Faculty of Science and Technology, University of Twente, P.O. Box 217, 7500 AE Enschede, The Netherlands.

E-mail: J.M.J.Paulusse@utwente.nl

Table of contents

Chemical structures of SCNP formation and functionalization S-2

DLS data S-2

^{1}H NMR spectra S-3

Zeta-potential data S-3

DLS data S-4

Flow cytometry data S-4

Confocal microscopy images S-5

Zeta-potential data S-5

UV-Vis data S-6

DLS data S-6

Confocal microscopy images S-7

Cytotoxicity S-7

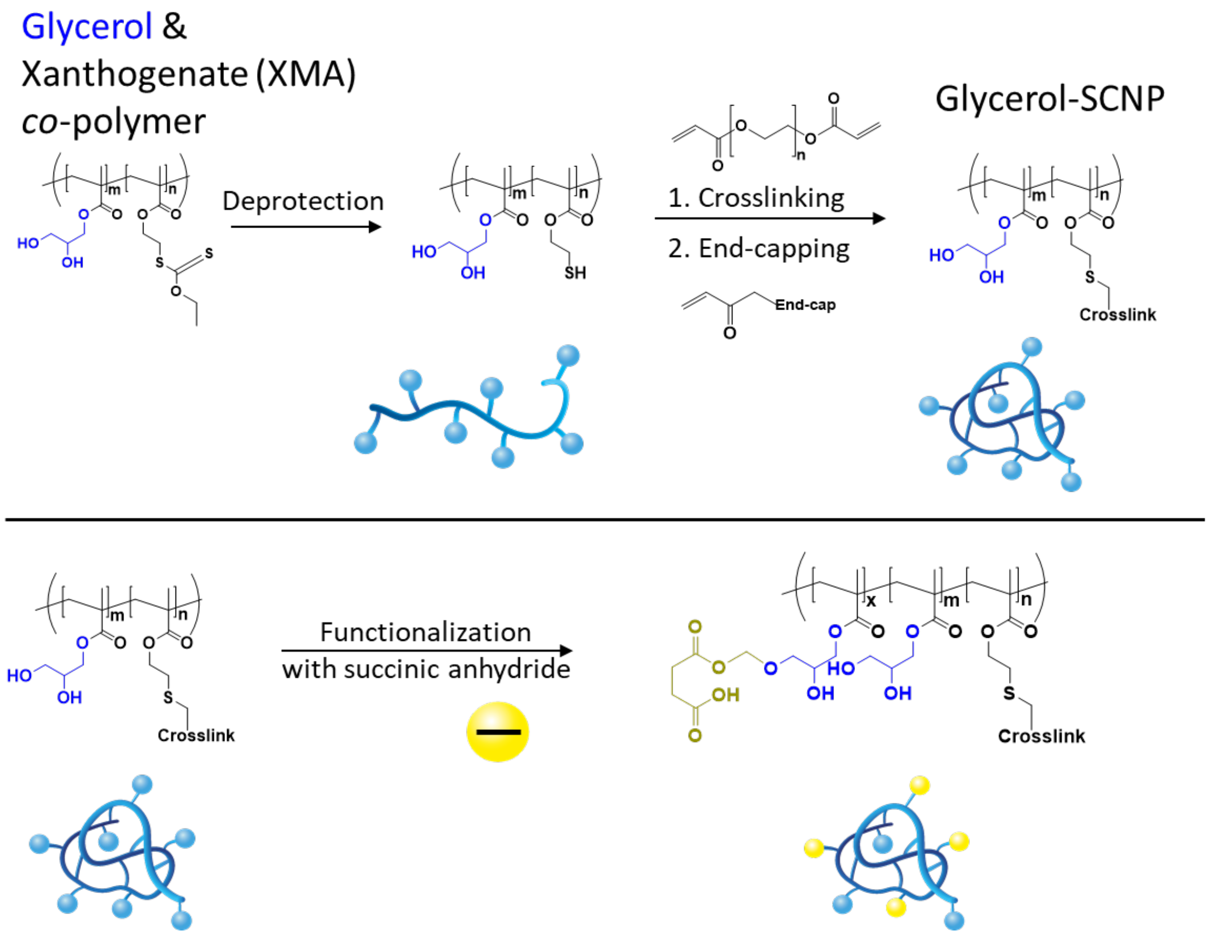

Figure S1. Synthesis of glycerol-SCNPs and further functionalization with succinic anhydride to equip SCNPs with negative surface charge.

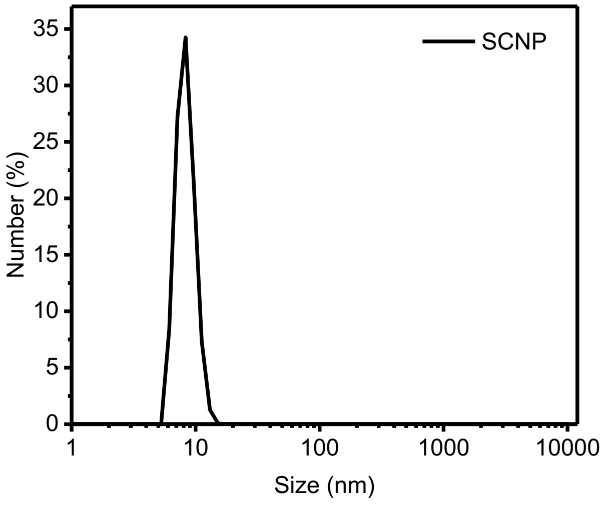

Figure S2. DLS of glycerol SCNP.

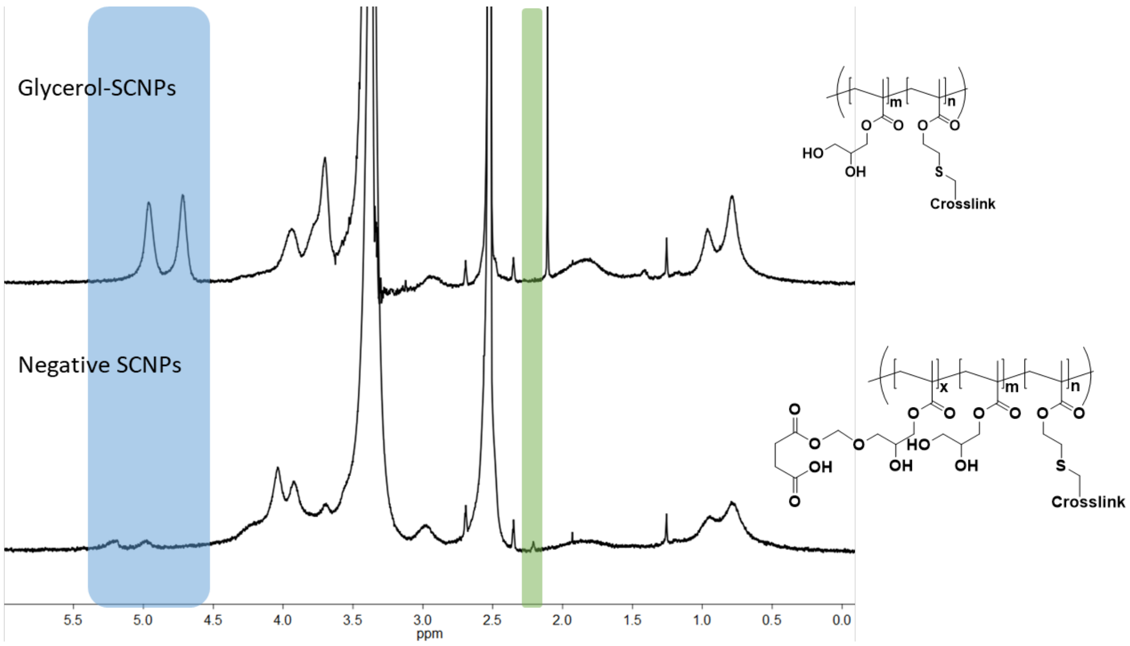

Figure S3. Glycerol functionalization with succinic anhydride supported by ^{1}H NMR. In blue the shift of signals from alcohol moieties on the SCNPs and in green an additional signal at 2.2 ppm for the negative SCNPs assigned to the formed succinate.

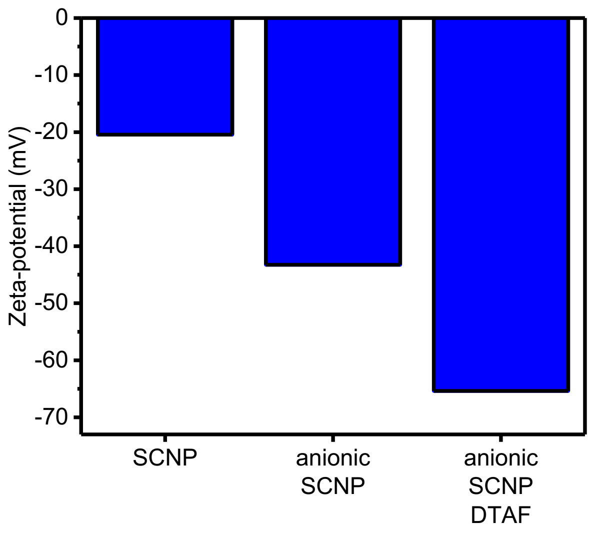

Figure S4. Zeta-potential of glycerol SCNPs and functionalized SCNPs.

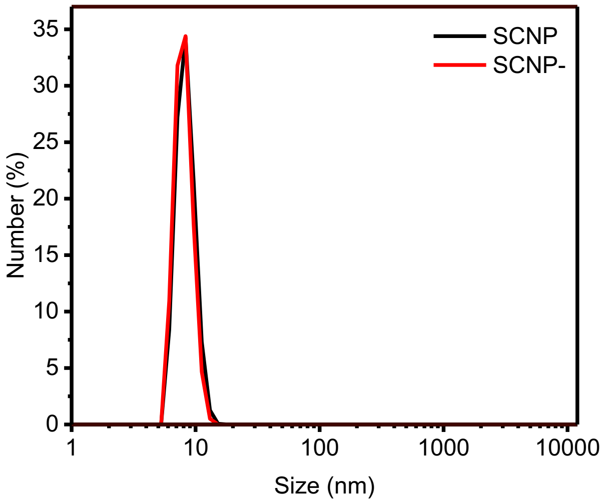

Figure S5. DLS of glycerol and anionic SCNPs; SCNP is 8.4 ± 1.3 nm and SCNP^{-} is 7.5 ± 1.7 nm.

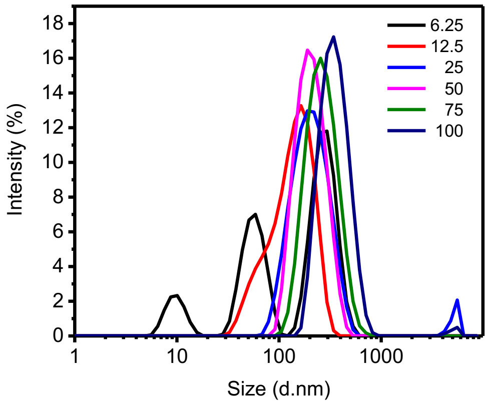

Figure S6. Intensity DLS plot of polyplexes at various polymer/SCNP ratios.

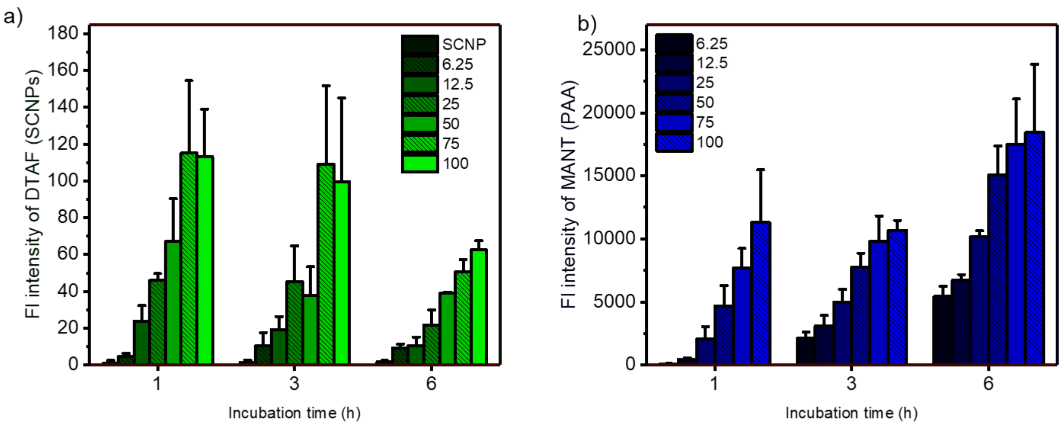

Figure S7. Fluorescent intensity of HeLa cells from a) SCNPs or b) pCBA-Abol signal measured by flow cytometry.

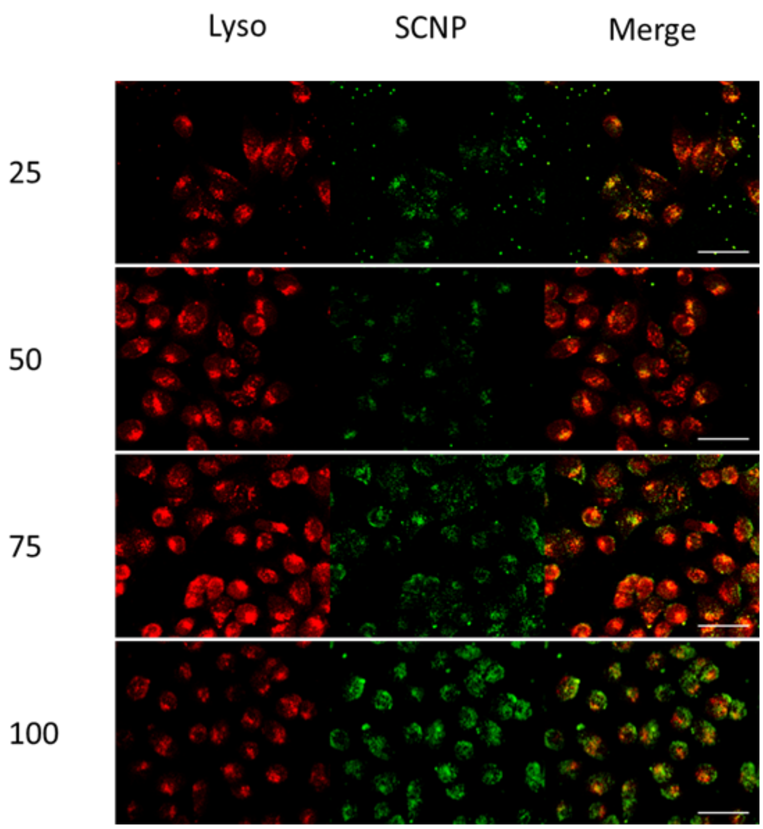

Figure S8. CLSM images of Hela cells incubated with polyplexes with selected w/w ratios to highlight the intracellular location

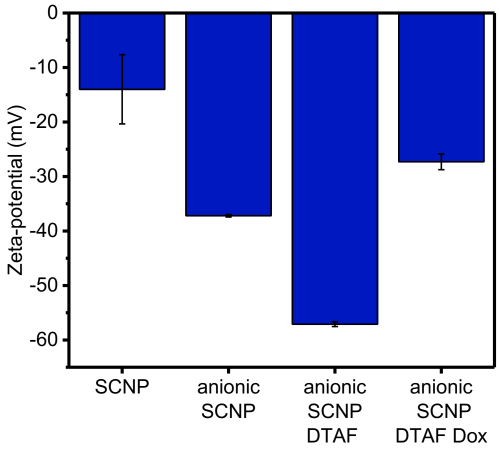

Figure S9. Zeta-potential of SCNPs and DOX loaded SCNPs

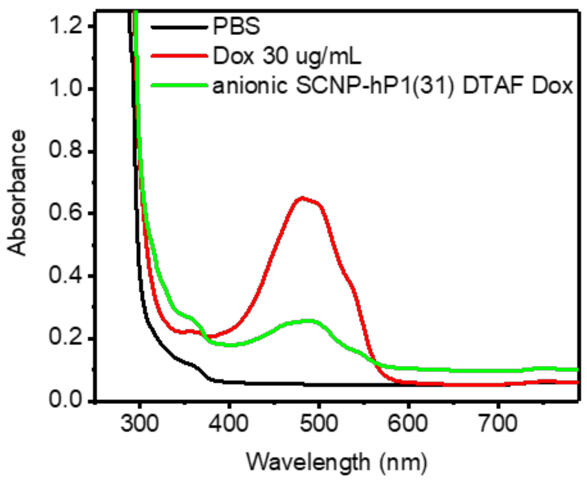

Figure S10. Absorbance of DOX loaded SCNPs in PBS (green) with reference PBS (black) and free DOX at 30 ug/mL (red) measured by UV-Vis.

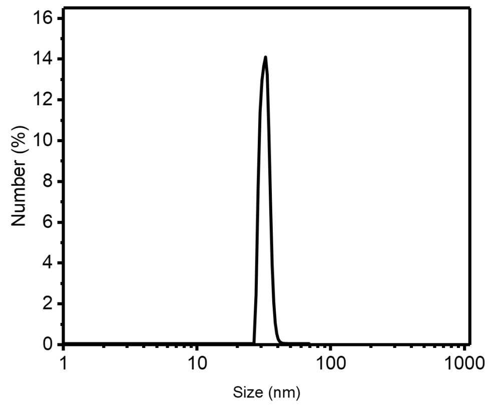

Figure S11. Size of DOX-SCNPs (39 nm ±14 nm) measured by DLS

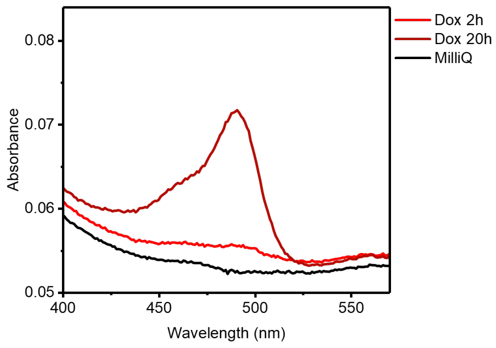

Figure S12. UV-Vis of DOX loaded SCNPs incubated in cell lysate for 2 or 20 h.

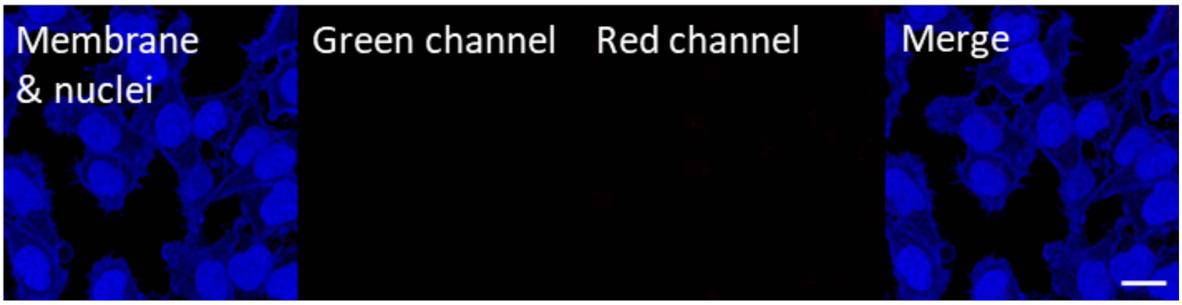

Figure S13. Confocal microscopy image of HeLa cells incubated with medium, the cell membrane and nuclei were stained in blue. The images were recorded with the same settings as the images in Figure 6.

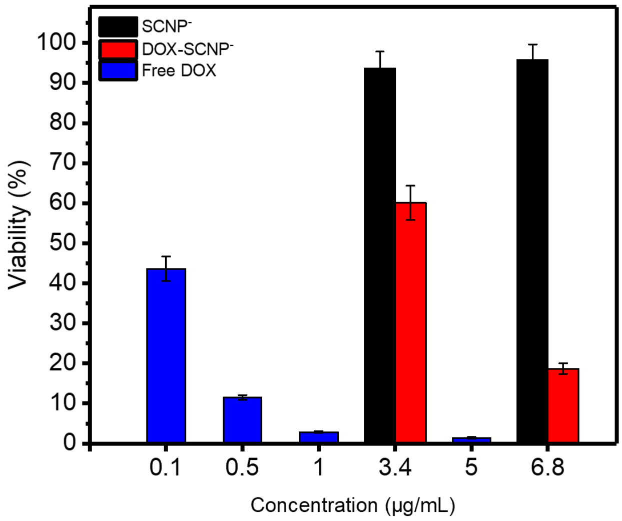

Figure S14. Viability of HeLa cells incubated with SCNPs, DOX-SCNPs or free DOX at corresponding DOX concentrations for 72 h.
